# Multiple Micronutrient Supplementation Using *Spirulina platensis* during the First 1000 Days is Positively Associated with Development in Children under Five Years: A Follow up of A Randomized Trial in Zambia

**DOI:** 10.3390/nu11040730

**Published:** 2019-03-29

**Authors:** Kazuya Masuda, Maureen Chitundu

**Affiliations:** 1 Institute of Economic Research, Hitotsubashi University, Tokyo 186-8603, Japan; 2 Programme Against Malnutrition, Lusaka P.O. Box 30599, Zambia; chitundu.maureen@gmail.com

**Keywords:** malnutrition, home-fortification, motor development, language skills, personal–social skills, Zambia, spirulina, the first 1000 days

## Abstract

Early childhood development relies on various micronutrients. We recently reported that home fortification of complementary foods using spirulina reduced the time to attain motor milestones in Zambian infants. The objective of this study is to estimate the long-term associations between spirulina supplementation during the first 1000 days and child gross motor development, fine motor development, language, and personal–social skills at preschool age. We used longitudinal data from a randomized trial conducted in Zambia. In 2015, 501 infants (age, 6–18 months) were provided daily supplements of maize-soy-based porridge with spirulina (SP) and without spirulina (CON). Supplementation period lasted for 16 months. In January 2018, children who participated in the initial trial were resurveyed (CON: 182 children; SP: 188 children; now aged 36–48 months). We assessed the infants’ gross motor development, fine motor development, language, and personal–social skills using a modified version of Malawi Development Assessment Tool. The initial clinical trial registration number was NCT03523182. Children in the SP group had higher scores in gross and fine motor development, language, and social skills than those in the CON group. Home fortification of complementary foods using spirulina during the first 1000 days improved development among Zambian children at preschool age.

## 1. Introduction

About 250 million children in developing counties are at risk of failing to meet development potential by the age of 5, and most of them live in Africa and South Asia [1,2]. Delayed development during childhood, especially in the first 1000 days, is associated with lower school performance, lower labor productivity, and lower income in later life [2,3]. Poor socioeconomic status (SES) in adulthood results in the risk of delayed development of offspring in the next generations, thus forming a vicious cycle. Therefore, the first 2 years of life provide a critical opportunity for interventions to break such intergenerational transmission of poor development and poverty.

An inexpensive and sustainable measure to tackle this issue is to utilize locally producible foods rich in multiple micronutrients (MMNs) as home supplements to complementary food. *Arthrospira platensis*, also known as spirulina, is a blue-green microalgae from the Oscillatoriaceae family, which is indigenous to Africa [4]. It contains a high percentage of protein, and is rich in MMNs that are known to support brain growth and development, such as beta-carotene, B vitamins, and minerals including calcium, iron, magnesium, manganese, potassium, and zinc [5,6,7,8,9,10]. The cost of producing spirulina is much lower than that of producing other comparably protein-rich foods, such as soya beans or beef [11], and therefore may potentially be a sustainable measure for meeting the nutritional demands of infants in low- to middle-income countries. 

Postnatal supplementation of spirulina has shown to reduce growth faltering of the children under the age of 5 in Zambia [12], Burkina Faso [13,14], and Palestine [15]. We recently reported that the 12-month spirulina supplementation of infants reduced the incidence of upper respiratory infection and improved motor development [16]. However, no study has ever tested if such positive effects of MMN supplementation are persistent or more evident after the non-intervention period.

This study aimed to assess the long-term effect of home fortification of complementary foods using spirulina on motor and mental development of preschool children in Zambia after a non-intervention period of 1.5 years. The testable hypothesis of the present study is fourfold; children who received spirulina in the initial trial had higher scores in gross motor skills, fine motor development, receptive and expressive language skills, and personal–social skills at follow-up after a non-intervention period of 18 months. We previously reported that spirulina supplementation in the initial trial reduced upper respiratory infection and time to walking unassisted [16]. The present study provides the results of the follow-up survey and the effects of the intervention on child development (secondary endpoint). The results of the primary endpoint (i.e., growth) and other outcomes (i.e., morbidity) were reported separately [17].

## 2. Materials and Methods 

### 2.1. Study Design

To evaluate the long-term effects of spirulina supplementation, the present study presented the findings from a follow-up survey. The initial trial was conducted in the Luapula Province of Zambia from May 2015 to April 2016 using an open-labeled randomized control trial (ClinicalTrials.gov registration#: NCT03523182) that combined a spirulina-fed treatment (SP) group and a control (CON) group. About 501 infants, aged between 6 and 18 months, were randomly fed with either: (1) maize-soy-based porridge with spirulina (SP); or (2) maize-soy-based porridge without spirulina (CON). The initial supplementation period was 12 months. However, after the study protocol was approved, we reserved the funding for another 4 months of supplementation and decided to continue supplementation from May 2016 to August 2016 with informed consent from the participants. Inclusion criteria were: (1) age between 6 and 18 months, (2) a singleton birth child, (3) residence in the study area, and (4) informed consent from at least one caregiver. Exclusion criteria included: (1) presence of severe illness warranting hospitalization on the enrolment session day, and (2) enrolment in any other clinical trial. Nutrient composition of the supplements are presented in the Supplementary Table S1.

**Figure 1:**
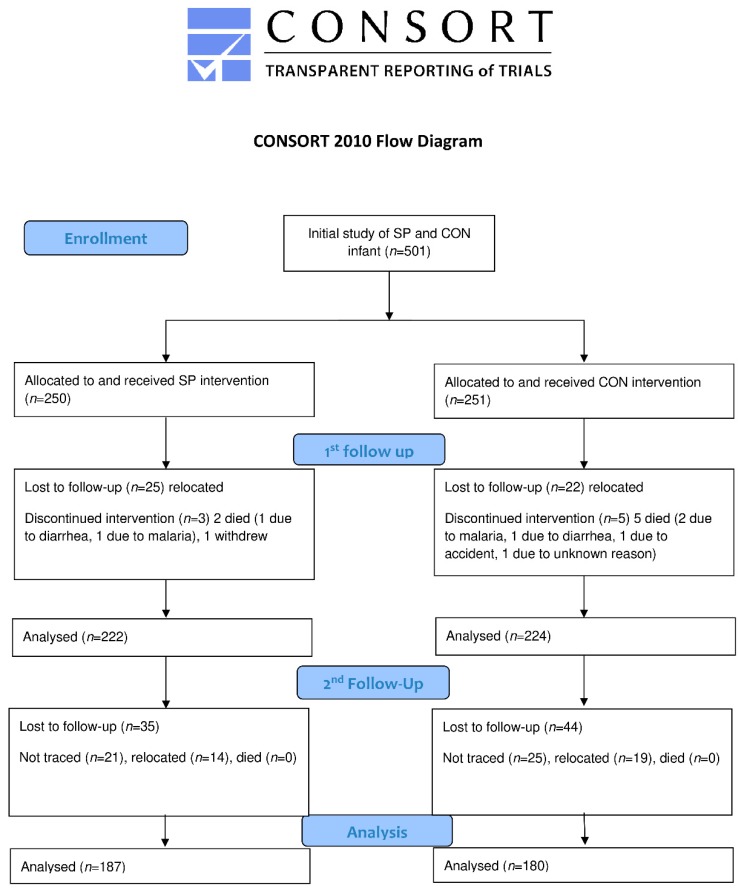
Flow chart of study participants. SP, maize-soya based control porridge plus the multiple micronutrient spirulina; CON, maize-soya based control porridge supplementation.

In January 2018, trained local health workers contacted all potential participants, and the households with infants who participated in the initial trial were located and invited to participate in the follow-up survey. Children were 36–48 months old at the time of the follow-up survey. A total of 370 (SP: 188; CON: 180) children attended the survey (Figure 1). 

The probabilities of the attrition were low and not different between the SP and CON groups. The attrition rates was not systematically associated with any of the baseline child or maternal characteristics (Supplementary Table S2). The final dataset involved 367 children (SP: 187; CON: 180). All 167 observations available in the follow-up survey were included to conduct intention-to-treat treatment analysis using the full analysis set.

### 2.2. Measurement

Assessment of child development included the evaluation of gross motor development, fine motor development, language skills, and personal–social skills. Motor development of children was assessed by adding the gross and fine motor subscale scores. Mental development was assessed by adding the language and personal-social subscale scores. Trained assistants, who were blinded to the study groups, visited the household and read all questions out loud to mothers or main caregivers. 

The two primary outcomes of the present study were motor development and mental development, which were evaluated using Malawi Developmental Assessment Tool (MDAT), with minor adaptations [18]. We assessed the children’s locomotor skills (6 items), eye–hand coordination (13 items), language skills (11 items), and personal–social skill development (8 items) (Supplementary Questionnaire S1). Each item was directly tested by a trained evaluator and scored as 1 if the child had performed one task or 0. The raw score of each subscale was calculated by summing the scores of that domain. The performance of the MDAT has been validated in several developing countries [18].

Furthermore, to isolate the treatment effects from the confounders in subsequent analysis, data on household SES and dietary habits were collected. SES parameters, such as parental demographic characteristics and household economic activity, were evaluated using a questionnaire-based interview. Dietary habits were assessed by obtaining data on food items that were fed to the child one week prior to the survey through a questionnaire-based interview (Supplementary Material S1). Because the dietary diversity of children in the treatment group and the control group was measured in the same month and in the same village, the relative differences among samples in terms of income level, parental nutritional knowledge, and child feeding practices were determined. The height and the weight of children were assessed at the baseline (Supplementary Material S1). Summary statistics at baseline and follow up of the developmental samples are shown in Table 1.

For the follow up study, we hypothesized that children in SP group would attain better scores on motor and mental development at preschool age than those in CON group. We estimated the effect size that can be detected by the present study based on the number of the full analysis set sample in the initial trial (*n* = 446), and assumed that the lost to follow up were not more than 20%. As a result, we expected that at least 365 children aged between 36 and 48 months would attend the follow up survey. With this sample size and a power of 0.80 and a two-sided significance level of 0.05, and with an equal division between SP and CON groups, we expected to be able to detect a difference of more than 0.29 SD between SP group and CON group. 

### 2.3. Ethical Statement

The study protocol (Supplementary Protocol S1) for initial trial was approved by the Biomedical Research Ethics Committee of the University of Zambia. This study secured voluntary participation and participant confidentiality throughout the study. The parents of participating infants provided informed consent.

### 2.4. Statistical Analysis 

Trained assistants entered data into an electronic database. We analyzed data using STATA15 software (StataCorp LLC, College Station, TX, USA). Raw scores of development measures were converted into z-scores (with 0 as mean and 1 as standard deviation) using the sample mean and standard deviation of the study sample.

To identify the effects of spirulina intake on child development, we used linear regression model to analyze the child development outcome. The results of the regression model were adjusted for child’s age in months, sex, camp of residence, and mother’s age, and education at baseline, number of household members, and number of children under the age of 5 at baseline, and we presented the Cohen’s d effect size for each outcome. 

We examined the effect modification on the motor and mental development scores based on the extent of malnutrition at baseline (using binary measure which distinguishes children who were moderately or severely stunted from those who were not stunted based on whether height for z-score was below or above −2 standard deviations compared with the World Health Organization Multicentre Growth Standards [19]) and dietary diversity score at baseline (using a binary measure which distinguishes children with less diverse food consumption from those with diverse food consumption based on whether the score is below or above the median). All observations available in the follow-up survey were included to conduct intention-to-treat treatment analysis using the full analysis set. In all the analyses, a *p*-value of <0.05 (two-sided test) was considered significant.

## 3. Results

**Figure 2:**
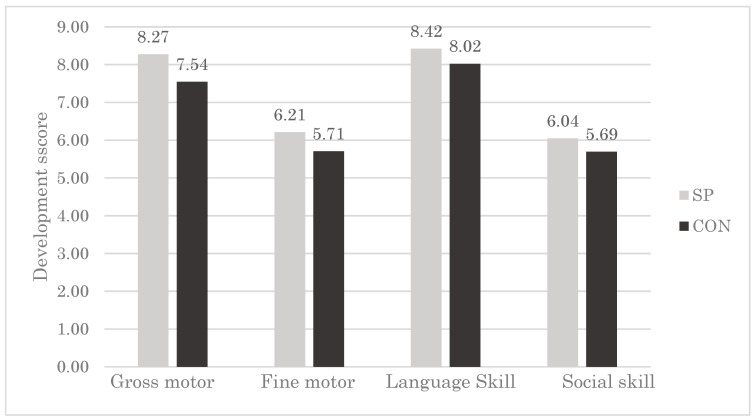
Gross motor development, fine motor development, language skills, and personal–social skills scores of children in each group. SP, maize-soya-based control porridge plus multiple micronutrient spirulina supplementation, which provides vitamins and minerals; CON, maize-soya-based control porridge supplementation. Gross motor scale, 0–10 fine motor; 0–8 language skills, 0–11; and personal–social skills, 0–8.

To study whether micronutrients from spirulina may assist the development even after a non-intervention period of 18 months, we compared the development score between the two groups using the modified version of MDAT. The raw score of motor development subscale was higher (by 0.73 points) in the SP group (8.27) than that in the CON group (7.54) (Figure 2). This difference between the two groups was observed even after assessing fine motor development, language skills, and personal-social skills. The percentage of children who failed to reach the motor development milestone was lower in the SP group (46.1%) than in the CON group (57.5%). Again, this difference across the groups was consistent for the domain of fine motor development, language skill development, and personal–social development (Figure S1).

To isolate treatment effects from confounders, linear regression was performed (Table 2). The results showed the significant differences in all domains of child development between the SP group and CON group (Table 2). Spirulina supplementation improved the children’s gross motor and fine motor skills (*p* < 0.01) as well as the language and personal–social skills (*p* < 0.05). Overall, the infants in the SP group obtained higher score in motor (Cohen’s d effect size: 0.42; 95% confidence interval (CI): 0.22, 0.63; *p* < 0.01) and mental development (Cohen’s d effect size: 0.32; 95% CI: 0.11, 0.52; *p* < 0.01) than those in the CON group. A heterogeneity analysis based on children’s height at baseline was conducted. Results showed that the across group difference in the motor development and mental development score were higher for children who were moderately or severely stunted at baseline (Height for Age Z score (HAZ) < −2) than non-stunted children (HAZ > −2). Similarly, the effects size on motor development was larger for children whose dietary diversity score at baseline was below median.

## 4. Discussion

The multiple micronutrient supplementation using spirulina in the first 2 years of life showed positive and lasting effects on child development even after 18 months of non-intervention period. We found consistent effects on all domains (gross motor, fine motor, language skill, and personal-social skill) of child development.

The observed effect size on motor development in the present study is comparable to or larger than those reported in the existing effectiveness trial in children in developing countries. In a study conducted in India, multiple micronutrient powder (MNP) was provided to households with children aged 6–18 months, and the intervention improved motor development in children aged 18 months with an effect size of 0.12 [20]. Another study in Bangladesh, which involved the administration of MNP to infants from 6 to 24 months of age, also reported that the MNP supplementation had a positive impact on motor development at 24 months, with an effect size of 0.15 [21]. Consistently, a cluster-randomized trial in Burkina Faso showed that provision of lipid-based nutrient supplements (LNSs) to children from 9 month to 18 months of age increased the motor development score by 0.34 standard deviations [22]. Although different assessment tools were used, it is noteworthy that our intervention improved motor development more than or at least as much as the LNS intervention in Burkina Faso, in which malaria and diarrhea treatments were added to the enhanced nutrition intervention. Another study in Pakistan consistently found the positive effects of MNP on motor development at 12-month, but failed to detect the significant effects of MNP on motor development at 24-month of age [23]. The possible explanation for this is that the effectiveness of MMN supplementation depends on the initial nutrition condition of the infants. Indeed, the proportion of moderately or severely stunted children at enrolment was lower in Pakistan (22%) than in Zambia (42%). 

Consistently, the improving effects of spirulina supplementation on motor development was evident in stunted children (HAZ < −0.2), and children whose dietary diversity score at baseline was below the median. By contrast, smaller effects were found in children who were not malnourished or had better dietary diversity at baseline. This result suggests that the MMN supplementation had larger effects on infants who were already disadvantaged and with higher risk of delayed motor development at enrolment. This result is consistent with those of other existing studies evaluating the effectiveness of MNP supplementation during the first 1000 days of child development in Burkina Faso, Chile, Bangladesh, and Indonesia [21,22,24,25]. In summary, this is the first study to present that the supplementation of MMN using locally producible foods, spirulina, improved motor development even after an 18-month non-intervention period.

Considering the context of Zambia, the results of the present study may suggest the generalizability of our findings to other nutritional interventions. For example, fortified blended flour, in which vitamins and minerals were added to a maize-based porridge, was distributed to malnourished children aged <5 years in Zambia [26,27], and may have the potential to produce similar positive effects on child development. The nutrient-rich nature of spirulina is optimally suited to reduce infant morbidity; however, as it contains MMN, determining the role of each micronutrient in improving child development is difficult. Thus, it is important for future studies to explore the mechanism through which spirulina intake may improve child development, and the generalizability to other nutrition interventions in this region.

The present study showed the positive effects of MMN supplementation using spirulina on expressive and receptive language development in children aged 36–48 months. Consistent with the results of the present study, a recent cluster randomized trial in Bangladesh, Pakistan, and Burkina Faso showed that LNS and MNP supplementation had positive effects on expressive language development in infants aged 18–24 months. A recent review reported that neurodevelopment related to language development occurs in the first 2–3 years of life [28,29], and thus the effects of supplementation becomes evident in children aged 2 years or older. The results of the present study suggest that such effects may remain persistent even at pre-school age after a 1.5-year non-intervention period.

Our data also showed that infants in the SP group attained higher score (SD: 0.26) on the personal–social skill subscale than those in the CON group. This finding is consistent with the results of the trial conducted in Burkina Faso and India, but contrary to those in Pakistan and Bangladesh. Again, the difference in the initial nutrient deficiency of the infants might play a role in this discrepancy. Indeed, the positive effects were only observed among children who were moderately or severely stunted at enrolment in the present study.

Our study had several limitations. First, we could not conduct the study in a blinded manner, and the mothers and assistants who delivered the porridge were aware of the treatment allocation details. However, group allocation was masked to the data collectors. Second, we assessed and reported the effectiveness of spirulina supplementation after an 18-month non-intervention period, and data between the endline survey and follow-up survey were inadequate, which may be another limitation. To validate the effectiveness of spirulina supplementation, an analysis using an inter-follow up survey at 9 months of non-intervention period, with a limited child development measure, is presented in Supplementary Table S3, and the results were consistent with the findings at 18 months. This further supports that spirulina supplementation consistently improved the child development during the post-intervention period. 

Lastly, the attrition was comparable to the other existing studies in this region, but might also be a limitation [30,31]. Such attrition is, however, unlikely to bias the results of the study. Our analysis showed that the baseline characteristics of the infants who were lost to follow-up were not different from the rest of the infants (Supplementary Table S1). This suggests that attrition occurred as if randomly and is unlikely to change the distribution of the baseline sample. Therefore, infants in the SP group and CON group in the full analysis set remained comparable. 

In summary, based on the findings of the present study, we conclude that fortification of complementary food with spirulina during the first 2 years of life had long-term beneficial effects on child development. Therefore, spirulina may be a cost-effective home-fortification agent to improve human capital in resource-poor countries. To validate whether the improving effects of spirulina supplementation on child development remain evident in school age, adolescence, and even after entering the labor market, the further research by following up this cohort is encouraged.

## Figures and Tables

**Table 1:** Baseline and follow up characteristics of children by intervention group.

	SP	CON
	(*n* = 187)	(*n* = 180)
**Child characteristics**		
Age at follow up (months)	43.0 ± 4.5	43.5 ± 5.0
Child female (%)	52.1	46.5
Stunting at baseline (%)	42.1	43.0
Underweight at baseline (%)	19.5	24.3
Wasting at baseline (%)	9.7	10.7
Dietary diversity score (0–7)	5.2 ± 1.0	5.2 ± 1.0
HIV-positive at baseline (%)	3.1	2.5
Child exclusively breastfed for 6 months (%)	89.5	90.1
Length of exclusive breastfeeding (months)	5.9 ± 0.5	5.8 ± 0.5
**Maternal characteristics**		
Maternal age at baseline (years)	28.1 ± 6.5	27.6 ± 7.5
Maternal height at baseline (cm)	152.5 ± 12.8	154.1 ± 10.6
Maternal weight at baseline (kg)	49.7 ± 7.3	49.4 ± 8.5
Maternal education at baseline (years)	6.1 ± 4.7	5.9 ± 4.3
**Household characteristics**		
Farmer (%)	61.7	69.1
Number of household members at baseline (persons)	5.8 ± 2.1	5.7 ± 2.5
Number of household members under the age of 5 at baseline (persons)	2.2 ± 0.9	2.2 ± 1.1
Households which had access to electricity at baseline (%)	1.1	1.1

Note: CON, control porridge group; SP, spirulina porridge group. The CON group received porridge with soya. The SP group received the same distribution plus spirulina. The values in the first and second columns show mean ± Standard Deviation.

**Table 2:** The effects of spirulina intervention on child development scores.

Outcome: Standardized z Score Measuring	Motor Development	Mental Development	Gross Motor Development	Fine Motor Development	Language Skill	Personal-Social Skills
**All children**						
Effect size	0.42 ***	0.33 ***	0.37 ***	0.38 ***	0.24 **	0.28 **
95% CI	(0.22, 0.63)	(0.12, 0.54)	(0.16, 0.58)	(0.18, 0.59)	(0.03, 0.45)	(0.06, 0.49)
**Children with HAZ < −2.0 at baseline (*n* = 141)**						
Effect size	0.57 ***	0.37 **	0.55 ***	0.46 ***	0.22	0.41 **
95% CI	(0.25, 0.89)	(0.04, 0.70)	(0.23, 0.88)	(0.12, 0.80)	(−0.11, 0.55)	(0.05, 0.77)
**Children with HAZ > −2.0 at baseline (*n* = 190)**						
Effect size	0.36 **	0.23	0.25 *	0.35 **	0.15	0.20
95% CI	(0.07, 0.64)	(−0.07, 0.52)	(−0.04, 0.54)	(0.07, 0.63)	(−0.12, 0.43)	(−0.11, 0.51)
**Children with dietary diversity score < median at baseline (*n* = 128)**						
Effect size	0.75 ***	0.41 **	0.66 ***	0.67 ***	0.34 **	0.27
95% CI	(0.40, 1.09)	(0.07, 0.76)	(0.27, 1.04)	(0.36, 0.98)	(0.03, 0.66)	(−0.09, 0.62)
**Children with dietary diversity score > median at baseline (*n* = 209)**						
Effect size	0.20	0.27 *	0.22	0.13	0.16	0.32 **
95% CI	(−0.07, 0.47)	(−0.01, 0.55)	(−0.06, 0.49)	(−0.14, 0.39)	(−0.12, 0.43)	(0.03, 0.61)

Note: 95% confidence intervals are in parenthesis. Motor development scale, 0–18; mental development, 0–19; gross motor, 0–10; fine motor, 0–8; language skills, 0–11; and personal–social skills, 0–8. Effect sizes were calculated with adjusted mean. All specifications control for individual characteristics: age in months, gender, length of exclusive breast feeding in months and mothers’ characteristics: age, years of education, and weight. *** Significance at the 1% level, ** Significance at the 5% level, and * Significance at the 10% level.

## Supplementary Materials

The following are available online at https://www.mdpi.com/2072-6643/11/4/730/s1. Supplementary Table S1: Nutrient composition of the soya and spirulina supplements used in this study.. Supplementary Table S2: Correlate of attrition with household characteristics at baseline. Supplementary Table S3: The effects of spirulina supplementation on child development at 9 month of non-intervention period.. Supplementary Protocol S1: Trial protocol. Supplementary material S1: Details of the dietary diversity measures. Supplementary Questionnaire S1: Motor Development and Language/Social Skill Survey.
