# Supplementary material for: Multiple Micronutrient Supplementation Using Spirulina platensis during the First 1000 Days is Positively Associated with Development in Children under Five Years: A Follow up of A Randomized Trial in Zambia
Source: Nutrients. 2019 Mar 29;11(4):730. doi: 10.3390/nu11040730 (PMC6520735; doi:10.3390/nu11040730)
Supplement: Supplementary file 1 [file nutrients-11-00730-s001.zip › nutrients-456617-suppl/Supplementary material S1 Measurement of the dietary diversity score.docx]

Supplementary material S1 Measurement of the dietary diversity score

Data on the food items that were fed to the child within the prior one week were collected by the trained assistant using the questionnaire-based interview during home visits at baseline and follow up to monitor changes in eating behavior. The mother or caregiver was asked to describe the food or drink that the child was given starting with the previous day (7th day) to the 1st day. Following the FAO guideline, all the food or drink purchased and consumed outside the home was excluded [1]. Following an earlier study in Africa [2], food items were classified into seven categories: starchy staples, legumes, dairy, meat/poultry/fish/eggs, vitamin A-rich fruit and vegetables, other fruits/vegetables, and oil/fat/butter. Based on the collected data, a dietary diversity score of 0–7 was calculated for each child. The score represented the number of food categories that were consumed during more than half of the week by the child.

**References**

1. Kennedy, G., Ballard, T., Dop, M. C. Guidelines for measuring household and individual dietary diversity. Rome: Food and Agriculture Organization of the United Nations; 2011.

2. Arimond, M, Ruel MT. Dietary diversity is associated with child nutritional status: evidence from 11 demographic and health surveys. J Nutr. 2004;134(10): 2579-2585.
