# Supplementary material for: Multiple Micronutrient Supplementation Using Spirulina platensis during the First 1000 Days is Positively Associated with Development in Children under Five Years: A Follow up of A Randomized Trial in Zambia
Source: Nutrients. 2019 Mar 29;11(4):730. doi: 10.3390/nu11040730 (PMC6520735; doi:10.3390/nu11040730)
Supplement: Supplementary file 1 [file nutrients-11-00730-s001.zip › nutrients-456617-suppl/Table S1.docx]

Supplementary Table S1

Nutrient composition of the soya and spirulina supplements used in this study.

| Vitamin/ mineral/ macro nutrients | Spirulina (10 g) | Soy (40 g): Control group | Spirulina 10 g + Soy 40 g: Treatment group |
| --- | --- | --- | --- |
| β-carotene (μg RE) | 1800 | 22 | 1822 |
| Vitamin B1 (mg) | 0.48 | 0.284 | 0.764 |
| Vitamin B2 (mg) | 0.39 | 0.1 | 0.49 |
| Vitamin B3 (mg) | 3.9 | 0.8 | 4.7 |
| Vitamin B6 (mg) | 0.09 | 0.184 | 0.274 |
| Vitamin E (mg) | 1.06 | 9.12 | 10.18 |
| Vitamin K (μg) | 222 | 13.6 | 235.6 |
| Folic acid (μg) | 7.3 | 88 | 95.3 |
| Calcium (mg) | 7.05 | 73.2 | 80.25 |
| Phosphorus (mg) | 92.1 | 216.4 | 308.5 |
| Iron (mg) | 8.33 | 2.44 | 10.77 |
| Sodium (mg) | 21 | 0.4 | 21.4 |
| Potassium (mg) | 152 | 720 | 872 |
| Magnesium (mg) | 27.8 | 92 | 119.8 |
| Zinc (mg) | 0.104 | 1.8 | 1.904 |
| Copper (mg) | 0.026 | 0.388 | 0.414 |
|  |  |  |  |
| Calories | 38.6 | 162 | 200.6 |
| Protein (g) | 69.4 | 13.48 | 82.88 |
| Total fat (g) | 0.82 | 7.16 | 7.98 |
| Total carbohydrate (g) | 1.27 | 13.56 | 14.83 |

Note: The detailed macronutrient and micronutrient composition of the porridge in the two groups was compiled based primarily on food composition data from the Zambia Food Composition Tables published by the Zambian National Food and Nutrition Commission [1], adding data from standard tables of food composition in Japan and FAO, and Siva et al. [2, 3, 4],

**References**

1. National Food and Nutrition Commission. Zambia food composition table-4^th^ edition. 2009. Lusaka, Zambia.

2. Siva Kiran RR, Madhu GM, Satyanarayana SV. Spirulina in combating Protein Energy Malnutrition (PEM) and Protein Energy Wasting (PEW)—a review. J Nutr Res. 2015;1: 62-79.

3. Watanabe T. Food composition tables of Japan and the nutrient table/database. J Nutr Sci Vitaminol. 2015;61(Supplement): S25-S27.

4. Woot-Tsuen WL, Busson F, Jardin C. Food composition table for use in Africa. United States Department of Health, Education and Welfare Nutrition Division. 1968;36.
