# Supplementary material for: Multiple Micronutrient Supplementation Using Spirulina platensis during the First 1000 Days is Positively Associated with Development in Children under Five Years: A Follow up of A Randomized Trial in Zambia
Source: Nutrients. 2019 Mar 29;11(4):730. doi: 10.3390/nu11040730 (PMC6520735; doi:10.3390/nu11040730)
Supplement: Supplementary file 1 [file nutrients-11-00730-s001.zip › nutrients-456617-suppl/Table S2.docx]

**S2 Table. Correlate of attrition with household characteristics at baseline**

| Outcome | 1 if attrited between baseline and follow up | |
| --- | --- | --- |
| Age of child in months | 0.00 | (-0.01, 0.01) |
| 1 if female | 0.04 | (-0.04, 0.12) |
| Number of household member | 0.02 | (-0.01, 0.04) |
| Number of under 5 member | -0.01 | (-0.06, 0.04) |
| ln(household consumption) | -0.01 | (-0.05, 0.03) |
| ln(household expenditure) | 0.01 | (-0.03, 0.05) |
| 1 if lives in Mansa | 0.05 | (-0.04, 0.13) |
| 1 if received any government assistance in last 12 months | -0.09 | (-0.28, 0.10) |
| 1 if with diarrhea | 0.05 | (-0.03, 0.13) |
| 1 if with fever | -0.03 | (-0.13, 0.07) |
| 1 if with cough | -0.02 | (-0.12, 0.08) |
| Mother's age | -0.00 | (-0.01, 0.00) |
| Mother's years of education | 0.00 | (-0.01, 0.01) |
| 1 if in treatment group | -0.04 | (-0.12, 0.04) |

Note: Values are estimated regression coefficients with 95% Cis in parenthesis.
 *** stands for significance at 1% level, ** at 5% level, and * 10% level.
