# Supplementary material for: Multiple Micronutrient Supplementation Using Spirulina platensis during the First 1000 Days is Positively Associated with Development in Children under Five Years: A Follow up of A Randomized Trial in Zambia
Source: Nutrients. 2019 Mar 29;11(4):730. doi: 10.3390/nu11040730 (PMC6520735; doi:10.3390/nu11040730)
Supplement: Supplementary file 1 [file nutrients-11-00730-s001.zip › nutrients-456617-suppl/Table S3.docx]

**S3 Table. The effects of spirulina supplementation on child development at 9 month of non-intervention period.**

| Outcome: Standardized z score measuring…. | Gross motor  development | Personal- social skill |
| --- | --- | --- |
| All children (N=323) |  |  |
| Effect size (mean) | 0.51*** | 0.27** |
| 95% CI | (0.29, 0.73) | (0.05, 0.49) |
|  |  |  |
| Children with HAZ<-0.2 at baseline (N=136) | | |
| Effect size (mean) | 0.66*** | 0.53*** |
| 95% CI | (0.30, 1.02) | (0.17, 0.89) |
| Children with HAZ>-0.2 at baseline (N=174) | | |
| Effect size (mean) | 0.39*** | 0.01 |
| 95% CI | (0.11, 0.67) | (-0.28, 0.30) |
|  |  |  |
| Children with dietary diversity score<median at baseline (N=119) | | |
| Effect size (mean) | 0.80*** | 0.76*** |
| 95% CI | (0.40, 1.21) | (0.38, 1.14) |
| Children with dietary diversity score>median at baseline (N=196) | | |
| Effect size (mean) | 0.32** | -0.02 |
| 95% CI | (0.06, 0.57) | (-0.30, 0.26) |

Note: 95% confidence intervals are in parenthesis. Motor development scale ranges from 0 to 7, and personal-social skill from 0 to 6. Effect sizes are calculated with adjusted mean. All specifications control for individual characteristics: age in months, gender, and mothers' characteristics: mother's age, and weight. *** stands for significance at 1% level, ** at 5% level, and * 10% level.
