# Supplementary material for: Multiple Micronutrient Supplementation Using Spirulina platensis during the First 1000 Days is Positively Associated with Development in Children under Five Years: A Follow up of A Randomized Trial in Zambia
Source: Nutrients. 2019 Mar 29;11(4):730. doi: 10.3390/nu11040730 (PMC6520735; doi:10.3390/nu11040730)
Supplement: Supplementary file 1 [file nutrients-11-00730-s001.zip › nutrients-456617-suppl/Supplementary Questionnaire S1.doc]

**Spirulina Follow up Survey in Zambia**

**Motor Development and Language/Social Skill Survey (January 2018)**

Alliance Forum Foundation, Japan (AFF) and Programme Against Malnutrition, Zambia (PAM)

| 1. | Household ID |  | 12. | Data Entered by (Officer ID) |  |
| --- | --- | --- | --- | --- | --- |
| 2. | Name of the Child |  | 13. | District Name |  |
| 3. | Household Head Name in 2015 |  | 14. | District Code  (See “District Code” in list) |  |
| 4. | Date of Interview  (Day/ Month/ Year) |  | 15 | Zone name |  |
| 5. | Name of Interviewer |  | 16 | Camp |  |
| 6. | Interviewer ID |  | 17. | Village |  |
| 7. | Date data checked  (Day/ Month/ Year) |  | 18. | Phone Number  Owned by Household Yes ☐ / No ☐ |  |
| 8. | Data checked by (Name) |  | 19. | Name of Main care taker |  |
| 9. | Data checked by (Officer ID) |  | 20. | Her/His Relationship to Child  (Fill in the code below)  1. Mother  2. Father  3. Grandmother  4. Grandfather | 5. Uncle / Aunt  6. Sibling  7. Other relatives  8. Other |
| 10. | Data Entered  (Day / Month/ Year) |  |  |  |  |
| 11. | Data entered by (Name) |  |  |  |  |

**Instruction to the Interviewers**

< Before the Interview>

1) Check the list of households you are going to visit today.

2) Input the HHID and the name of the study Child for the households you are going to visit today to ALL Answer Sheets.

3) Confirm the HHID and Name of the Child in the Answer Sheet with the Food and Nutrition Officer before heading to the households.

4) Make sure you have the Code List and the Answer Sheets before heading to the households.

< During the Interview>

1) For answers you have to choose from the code list, circle the number of the code firmly, or write the code number CLEARLY.

2) For any other questions, you can use the following codes for answers where applicable. **DO NOT leave any question blank.**

-9 = Not applicable

-8 = Don’t know

-7 = Refuse to answer

-6 = Don’t recall

-5 = Missing Data

< After the Interview>

1) The Food and Nutrition Officer should check together with the interviewer that he or she visited all the households required.

2) The Food and Nutrition Officer should review all the pages of the answer sheets and make sure there are no unanswered questions.

**Instruction to the Interviewers For【Motor Development and Language/Social Skill Survey】**

< Before the Interview >

Some questions need to be asked to the child directly for this survey (not through his/her parents). Here’s some important points to build a good relationship with the child to do an accurate survey.

- Smile and talk friendly to the child to make him/her feel relaxed with this interview.
- Child should sit together with his/her parents while you are interviewing his/her parents so that he/she gets used to being with you.
- **Explain to parents that they can’t help the child answer/ act for accurate survey.**
- If he/she seem to be too nervous, take some time to be friend with or give a toy (doll/car/picture book) and let him/her play with it while you are interviewing his/her parents.

< During the Interview >

- Only wait for the estimated time for the child to answer the question. Follow the estimated time. (e.g. [wait maximum 10 seconds.])
- Avoid telling the child whether the answer/action was right or wrong. Just say “Good boy/girl. Thank you” and move on to the next question.
- Parents should not interfere in the interview. (can be sit together)

**【Tool Check List】**

- Ball	(tennis ball size)
- Ball	(soccer ball size)
- i. Broom	(copy of larger one)
- ii. Matchbox
- iii. Plastic bottle (e.g. for oil)
- iv. Plate	(plastic from market)
- v. Cup	(plastic from market)
- vi. Spoon	(plastic from market)
- vii. Soap
- viii. Pencil or ball point pen
- ix. Coin	(any type)
- x. Car	(made of wood or plastic from market)
- Blocks	(square one inch size)
- Chiponde (peanut butter) (bottle or other plastic bottle with easy screw top)
- 4 Plain paper	(to write on with chalk/pencil 2 for you 2 for the child)
- 1 Plain paper	(A4 size to jump over)
- Sticks of two different lengths
- Basket	(for putting items in and for throwing balls into)
- Beads	(found in market as used to put in hair)
- String	(1 metre long. could be shoe lace. Tie the knot at the end)
- Stop watch

**Section 2-e-1. Fine Motor** Skills

| Household ID |  | Name of Child |  |
| --- | --- | --- | --- |

| Is this child present at the interview?  **1=Yes**  **0=No** | Can this child Throw a ball into a basket  (at least 1 of 3 times) 1 metre away?  **1=Yes**  **0=No**  (Stands 1 metre away from examiner’s basket and is able to throw the ball in on at least 1 of 3 occasions when it is tried. Demonstrated by examiner first. Can use the metre-long string to measure this.) | | | Can this child Run and kick a ball (soccer ball size) some distance?  **1=Yes**  **0=No**  (Able to run up to the ball and kick it well a good distance with a good swing forward of the leg and balancing on one foot.) | Can this child Jump over a piece of paper (widthways)?  **1=Yes**  **0=No**  (Put a piece of the examination paper (A4 size) on the ground. Ask the child to stand close to the side of the paper with both feet together and to jump over the less wide part of the paper and landing with both feet together. Can be demonstrated. Both feet need to come off the ground.) | Can this child Throw a ball up in the air and catch it with 2 hands? (at least 1 of 3 times)  **1=Yes**  **0=No**  (Can throw the ball up a good distance and catch it with two hands together.) | | | How many blocks can this child Tower? -up to 6 blocks  **0 ~ 6**  **If 4 blocks, write “4”**  (Show how to build a tower of blocks, and see if the child will copy you. Count how many you get up to 6 blocks. the blocks should laydown.) | Can this child Unscrew and screw back on the cap of the Chiponde bottle within 1 minute?  **1=Yes**  **0=No**  (Is able to actually screw and unscrew the lid off and on the chiponde bottle, not just pulling it off.) |
| --- | --- | --- | --- | --- | --- | --- | --- | --- | --- | --- |
| **F1** | **F2** | | | **F3** | **F4** | **F5** | | | **F6** | **F7** |
|  |  | 1^st^ |  |  |  |  | 1^st^ |  |  |  |
|  |  | 2^nd^ |  |  |  |  | 2^nd^ |  |  |  |
|  |  | 3^rd^ |  |  |  |  | 3^rd^ |  |  |  |

**Section 2-e-1. Fine Motor** Skills

| Household ID |  | Name of Child |  |
| --- | --- | --- | --- |

| Can this child Put 6 hair beads on to a shoe lace within 1 mins?  **1=Yes**  **0=No**  (See if the child is able to thread 6 hair beads on to a thread within 1 min.) | Can this child Choose the longest stick all the 3 times?  **1=Yes**  **0=No**  (Put down 2 sticks of different length on the ground and ask the child “which one is longest?” Do not indicate whether the child has done the correct response, but put him/her down again and ask the same question. See if the child can do it 3 times.) | Can this child make a bridge with blocks?  **1=Yes**  **0=No**  (Make a bridge for the child out of 3 blocks and see if the child can do the same. Keep yours up while the child is doing it and see if the child will copy you.) | Can this child Copy a circle (needs to be completed) on a paper? (could be with chalk or in the sand with a stick)  **1=Yes**  **0=No**  (Draw a circle and see if she can copy you. Can do anything that is near to a circle that is complete or nearly complete. Continuous spiral motions as in scribbling do not count.) | Can this child Copy a cross on a paper? (could be with chalk or in the sand with a stick)  **1=Yes**  **0=No**  (Draw a cross and see if the child can copy you. Anything where the two lines intersect counts. The two lines do not need to be the same size and can be at any angle. Can use back of questionnaire.) | Can this child Draw a square/rectangle on a paper? (could be with chalk or in the sand with a stick)  **1=Yes**  **0=No**  (Draw a square and see if the child can copy you. Must have four sides and four sharp corners, but does not need to be perfect. Can use back of questionnaire.) |
| --- | --- | --- | --- | --- | --- |
| **E8** | **F9** | **F10** | **F11** | **F12** | **F13** |
|  |  |  |  |  |  |

**Section 2-e-2. Motor Development**

| Household ID |  | Name of Child |  |
| --- | --- | --- | --- |

| Is this child present at the interview?  **1=Yes**  **0=No** | Can this child Kneel (as in a respectful way) and get up without using hands?  **1=Yes**  **0=No**  (Able to get down on to his knees staying in an upright position and then get up without using hands. **The child can not touch the ground or any parts of his/her body.**) | Can this child Jump with feet together off the ground?  **1=Yes**  **0=No**  (Able to jump with both feet leaving the floor together. Needs to get both feet off the ground.) | How long can this child Stand on one foot? – up to a minute  **0 second ~ 1 minute**  **If 10 seconds, write “10 seconds”**  (Ask child to raise one foot usually by bending his knee and maintain a good balance on his other foot) | How many steps can this child Walk on heels?  **Write in number**  ***e.g. I*f 3 steps, Write “3”**  (Can walk on heels easily. The child must try twice.) | | | How many steps can this child Walk on tip toes?  **Write in number**  ***e.g. I*f 3 steps, Write “3”**  (Able to balance on tip toes and walk keeping up on the toes well with good balance. The child must try twice.) | | | How many times can this child Hop on one foot without support?  **Write in number**  ***e.g. I*f 3 steps, Write “3”**  (Makes consecutive hops with the same leg raised throughout. Can be demonstrated by the examiner first. The child must try twice.) | | |
| --- | --- | --- | --- | --- | --- | --- | --- | --- | --- | --- | --- | --- |
| **E9** | **E10** | **E11** | **E12** | **E13** | | | **E14** | | | **E15** | | |
|  |  |  |  |  | 1^st^ |  |  | 1^st^ |  |  | 1^st^ |  |
|  |  |  |  |  | 2^nd^ |  |  | 2^nd^ |  |  | 2^nd^ |  |

**Section 2-e-3. Language skills**

| Household ID |  | Name of Child |  |
| --- | --- | --- | --- |

| How many of the following body parts can this child Point to (legs, ears, nose, eyes)?  **0~4**  (eg. “Where is your nose?) | Can this child Say his or her name?  **1=Yes**  **0=No**  (Ask his/her name. Passes if can say his/her first name.) | How may can this child Name objects in the basket?  **Number of items he/she names correctly up to 10**  **(if able to name 3 items, mark “3”.)**  (Ask“What is this?” Give the child all 10 items and see how many he or she can do. Answer the number of items he/she name. Doesn’t matter which ones.) | How many does this child Know actions of objects?  e.g. “which one do you use for sweeping?” Which one is for drinking?  **Number of items he/she answer correctly up to 10**  **(if able to name 3 items, mark “3”.)**  (Put out a few different objects from the basket and ask questions above or similar ones and see if the child can point to the right objects.) | How many can this child Identify objects (can give you objects you have named) in the basket – at least 10?  **Number of items he/she identify correctly up to 10**  **(if able to name 3 items, mark “3”.)**  (Say to the child “Give me the spoon, cup...etc”. Count how many out of 10 the child has done. Make sure at least 10 objects are put out. Answer YES if child can identify at least 10 of the ten objects.) | Can this child understand 5 prepositions and follow tasks related to this? Put the beads in the cup, put it on the cup, put it next to the cup, put it behind the cup, and put it in front of the cup?  **1=Yes**  **0=No**  (“behind “ “in front of”and ”next to “can be done with cup and a person.) | Can this child count up to 3?  **1=Yes**  **0=No**  (Put 3 blocks on the table and ask the child. How many are there? Answer YES to this if he/she can count 3.) | Can this child count up to 5?  **1=Yes**  **0=No**  (Put 5 blocks on the table and ask the child. Can you count these? How many are there? Answer YES to this if he/she can count 5.) |
| --- | --- | --- | --- | --- | --- | --- | --- |
| **L1** | **L2** | **L3** | **L4** | **L5** | **L6** | **L7** | **L8** |
|  |  |  |  |  |  |  |  |

**Section 2-e-4. Social skills**

| Household ID |  | Name of Child |  |
| --- | --- | --- | --- |

| Can this child Go to the toilet by him/herselves?  **2=Yes (on the toilet/pit latrine)**  **1=Yes (but anywhere.** **not necessarily on the toilet)**  **0=No**  (Can go and do a poo or a pee by him/herselves without help.) | Can this child Eat food with seeds in it or bones?  **1=Yes**  **0=No**  (Is able to eat for example fish with bones in it or maybe tangerines with seeds. answer yes if can do one of these) | Can this child Wash hands well by him/herselves before/after eating?  **1=Yes**  **0=No**  (Is able to wash his or her hands without any help before and after eating.) | Can this child Dress by him/herselves? (trousers/skirt, shirt/T-shirt)  **2=Yes (completely)**  **1=Yes (not completely)**  **0=No**  (I2: help only tying shoelaces, buttoning or zipping is “2.”1: put on at least one article of clothing, for example a T-shirt or skirt.) | |
| --- | --- | --- | --- | --- |
| **S1** | **S2** | **S3** | **S4** | |
|  |  |  | trousers/  skirt |  |
|  |  |  | shirt/  T-shirt |  |

**Section 2-3,4. Language and Social skills【Script L1-L8】**

**L1**

| **Tool** | **Script** | **Time you wait** |
| --- | --- | --- |
|  | You: Where is your nose? Can you show me pointing your nose? |  |
|  | Child: (- Showing his/her answer -) | [10 seconds max] |
|  | You: Thank you. How about ears? Where is your ears? [You can switch to “mouth” if the child point ears with the previous question.] |  |
|  | Child: (- Showing his/her answer -) | [10 seconds max] |
|  | You: Thank you! |  |
|  | **Mark: “1” if able to do 2 body parts, “0” 1 body parts or less** |  |

**L2**

| **Tool** | **Script** | **Time you wait** |
| --- | --- | --- |
|  | You: What is your name? |  |
|  | Child: (- Answering his/her answer -) | [10 seconds max] |
|  | [If he/she don’t say anything, You can ask “Can you tell me your name?” additionally once.] |  |
|  | You: Thank you! |  |
|  | **Mark: “1” if Yes, “0” if No** |  |

L3

| **Tool** | **Script** | **Time you wait** |
| --- | --- | --- |
| - basket  - 10 items (i~x) | [put 10 items into the basket. Try not to show the items before test] |  |
|  | You: What is this? [put out one items from the basket] |  |
|  | Child: (- Answering his/her answer -) | [10 seconds max] |
|  | [If he/she don’t say anything, You can ask “Can you tell me the name of this?” additionally once.] |  |
|  | -repeat until you show 10 items.- |  |
|  | You: Thank you! |  |
|  | **Mark: Number of items he/she name correctly (if able to name 3 items, mark “3”. up to “10”)** |  |

**Section 2-e-3,4. Language and Social skills【Script L1-L8】**

L4

| **Tool** | **Script** | **Time you wait** |
| --- | --- | --- |
| - basket  - 10 items (i~x) | [put out “broom” “spoon” and “car” from the basket.] |  |
|  | You: Which one do you use for sweeping? |  |
|  | Child: (- Answering his/her answer -) | [10 seconds max] |
|  | [put out “cup” “matchbox” and “pencil” from the basket] |  |
|  | You: Which one do you use for drinking? |  |
|  | Child: (- Answering his/her answer -) | [10 seconds max] |
|  | You: Thank you! |  |
|  | **Mark: “1” if able to do more than one of it, “0” Neither** |  |

L5

| **Tool** | **Script** | **Time you wait** |
| --- | --- | --- |
| - basket  - 10 items (i~x) | [put out all 10 items from the basket.] |  |
|  | You: Give me the Spoon. |  |
|  | Child: (- Answering his/her answer -) | [10 seconds max] |
|  | [If he/she don’t do anything, You can ask “Can you give me the spoon?” again once.] |  |
|  | You: Thank you! Can you give me the Plate. |  |
|  | - repeat until you ask 10 items.- |  |
|  | You: Thank you! |  |
|  | **Mark: Number of items he/she identify correctly (if able to name 3 items, mark “3”. up to “10”)** |  |

**Section 2-e-3,4. Language and Social skills【Script L1-L8】**

L6

| **Tool** | **Script** | **Time you wait** |
| --- | --- | --- |
| -cup  -bean | You: Can you put the bean UNDER the cup? |  |
|  | Child: (- Answering his/her answer -) | [15 seconds max] |
|  | [If he/she don’t do anything, You can ask “Can you put the bean under the cup?” again once.] |  |
|  | You: Can you put the bean ON the cup? [Same as above] |  |
|  | You: Can you put the bean NEXT to the cup? [Same as above] |  |
|  | You: Can you put the bean BEHIND the cup? [Same as above] |  |
|  | You: Thank you! |  |
|  | **Mark: “1” if able to do more than 3, “0” 2 or less** |  |

L7

| **Tool** | **Script** | **Time you wait** |
| --- | --- | --- |
| -3 blocks | [put 3 blocks on the table or on the floor] |  |
|  | You: How many are these? Can you count it? |  |
|  | Child: (- Answering his/her answer -) | [15 seconds max] |
|  | You: Thank you! |  |
|  | **Mark: “1” if Yes, “0” if No** |  |

L8

| **Tool** | **Script** | **Time you wait** |
| --- | --- | --- |
| -5 blocks | [put 5 blocks on the table or on the floor] |  |
|  | You: How many are these? Can you count it? |  |
|  | Child: (- Answering his/her answer -) | [20 seconds max] |
|  | You: Thank you! |  |
|  | **Mark: “1” if Yes, “0” if No** |  |
